# Schistosome Sulfotransferases: Mode of Action, Expression and Localization

**DOI:** 10.3390/pharmaceutics14071416

**Published:** 2022-07-06

**Authors:** Meghan A. Guzman, Anastasia Rugel, Sevan N. Alwan, Reid Tarpley, Alexander B. Taylor, Frédéric D. Chevalier, George R. Wendt, James J. Collins, Timothy J. C. Anderson, Stanton F. McHardy, Philip T. LoVerde

**Affiliations:** 1 Department of Microbiology and Immunology, University of Texas Health, San Antonio, TX 78229, USA; ruedam1@uthscsa.edu (M.A.G.); rugel.j@hotmail.com (A.R.); 2 Department of Biochemistry and Structural Biology, University of Texas Health, San Antonio, TX 78229, USA; alwan@uthscsa.edu (S.N.A.); taylorab@uthscsa.edu (A.B.T.); 3 Center for Innovative Drug Discovery, University of Texas at San Antonio, San Antonio, TX 78249, USA; chancesjigs@mindspring.com (R.T.); stanton.mchardy@utsa.edu (S.F.M.); 4 Host Pathogen Interactions Program, Texas Biomedical Research Institute, San Antonio, TX 78227, USA; fcheval@txbiomed.org; 5 Department of Pharmacology, UT Southwestern Medical Center, Dallas, TX 75390, USA; george.wendt@utsouthwestern.edu (G.R.W.); jamesj.collins@utsouthwestern.edu (J.J.C.III); 6 Disease Intervention & Prevention, Texas Biomedical Research Institute, San Antonio, TX 78227, USA; tanderso@txbiomed.org; 7 Department of Pathology and Laboratory Medicine, University of Texas Health, San Antonio, TX 78229, USA

**Keywords:** schistosomiasis, *Schistosoma mansoni*, *S. haematobium*, *S. japonicum*, oxamniquine derivatives, whole worm in situ hybridization, mechanism of killing

## Abstract

Oxamniquine (OXA) is a prodrug activated by a sulfotransferase (*SULT*) that was only active against *Schistosoma mansoni*. We have reengineered OXA to be effective against *S. haematobium* and *S. japonicum*. Three derivatives stand out, CIDD-0066790, CIDD-0072229, and CIDD-0149830 as they kill all three major human schistosome species. However, questions remain. Is the OXA mode of action conserved in derivatives? RNA-interference experiments demonstrate that knockdown of the *SmSULT*, *ShSULT*, and *SjSULT* results in resistance to CIDD-0066790. Confirming that the OXA-derivative mode of action is conserved. Next is the level of expression of the schistosome SULTs in each species, as well as changes in *SULT* expression throughout development in *S. mansoni*. Using multiple tools, our data show that *SmSULT* has higher expression compared to *ShSULT* and *SjSULT*. Third, is the localization of *SULT* in the adult, multicellular eucaryotic schistosome species. We utilized fluorescence in situ hybridization and uptake of radiolabeled OXA to determine that multiple cell types throughout the adult schistosome worm express *SULT*. Thus, we hypothesize the ability of many cells to express the sulfotransferase accounts for the ability of the OXA derivatives to kill adult worms. Our studies demonstrate that the OXA derivatives are able to kill all three human schistosome species and thus will be a useful complement to PZQ.

## 1. Introduction

Previous treatments for schistosomiasis have consisted of a multitude of drugs, many of which have fallen out of favor in subsequent years, due to resistance, effectiveness, cost, and side effects [1,2]. Oxamniquine (OXA) was an efficient drug for treating *Schistosoma mansoni* infections but was ineffective against the other two main schistosome species infecting humans, *S. haematobium* and *S. japonicum*. Because of this species specificity, OXA was the drug of choice in Brazil [3], where only *S. mansoni* is present. However, resistance has developed in the field [4,5,6,7] and was selected for use in the laboratory [8,9]. Praziquantel (PZQ) was developed around the same time as OXA but had the advantage of treating infections caused by all three schistosome species. PZQ has replaced OXA as a schistosomicide in all parts of the world, because of its broader spectrum and effectiveness [10]. PZQ has few adverse side effects and is extremely cost-effective, due to an expired patent. However, PZQ is not effective against immature parasites [11,12,13]. There is some concern that the emergence of PZQ-resistant worms would be inevitable due to the use of mass chemotherapy of PZQ, adding selection pressure to the parasite populations [14]. Evidence for resistance to PZQ has already been observed in the field and selected for in the laboratory [14,15,16,17,18,19]. The efficacy of OXA and PZQ is comparable, although in some cases OXA is more effective against *S. mansoni* when PZQ drug failure is observed [20].

Previous studies have identified both the mechanism of OXA activity and the mechanism for OXA resistance [21]. OXA is a prodrug that is enzymatically activated in the parasite [21,22]. OXA binds to a specific *S. mansoni* sulfotransferase, known as *SmSULT*-OR, where it is transiently sulfated, resulting in its activation [23]. Sulfotransferases are enzymes that catalyze the transfer of a sulfuryl group (SO_3_) from a sulfate donor, such as 3′-phosphoadenosine 5′-phosphosulfate (PAPS), to substrates. Activated OXA binds to DNA and other macromolecules, resulting in the killing of the schistosome adult worms [21,23,24,25]. This affects both adult sexes but mainly males, causing the parasite to detach from the hepatoportal circulation and move into the liver where they are eliminated. Mutations in the *S. mansoni* sulfotransferase (*SmSULT*) are responsible for OXA resistance both in the field and in lab-derived resistant isolates [6,7,21]. In a previous study, we identified an OXA derivative, CIDD-0066790, that would kill 85–100% of *S. mansoni*, 40% *S. haematobium* and 83% *S. japonicum* [26]. Using CIDD-0066790, in this paper, we address the question of whether the mechanism of killing for OXA derivatives that kill the three major human species of *Schistosoma* is the same as for OXA. We examine the relative abundance of sulfotransferases in *S. mansoni*, *S. haematobium*, and *S. japonicum*. In addition, we localize the distribution of *SULT* within the adult male schistosome tissues. The results address questions of relative abundance of *SULTs* between species, as well as identify the cells that express *SULTs* and how this contributes to the killing of a multicellular eukaryotic parasite.

## 2. Materials and Methods

### 2.1. Schistosome Parasites and Animal Infections

*Schistosoma mansoni*, *S. haematobium* and *S. japonicum* were maintained in the laboratory in *Biomphalaria glabrata*, *Bulinus truncatus,* and *Oncomelania hupensis* snails, respectively [27]. Cercariae collected from infected snails were used to infect male Golden Syrian hamsters in accordance with an IACUC protocol (UTHSCSA IACUC Protocol #20110087AR).

### 2.2. Parasite Harvesting

Once the schistosomes developed into the necessary stage of worms in the mammalian host (20–90 days depending on the schistosome species and stage of development) the animals were sacrificed in accordance with IACUC protocol (UTHSCSA IACUC Protocol #20110087AR) by intraperitoneal injection using Fatal-Plus (Butler Animal Health, Dublin, OH, USA), a sodium pentobarbital solution, with 10% heparin added. Adult worms were collected by perfusion [28]. In the case of *S. haematobium*, the worms were also manually dissected out of the mesenteries and fat deposits of the bowels using forceps. The adult worms were placed in culture in media containing 1X Dulbecco’s Modified Eagle Medium (DMEM, Gibco) and 1X antibiotic/antimycotic (Ab/Am, GIBCO). Three-hour schistosomula were mechanically transformed from cercariae according to [27].

### 2.3. RNA Extraction

Total RNA was obtained from frozen samples of adult *S. mansoni*, *S. haematobium*, and *S. japonicum* worms. All frozen samples were thawed on ice in RNAzol^®^ RT (Molecular Research Center Inc., Cincinnati, OH, USA) and sonicated (QSonica™, Newtown, CT, USA) for 1 s 10× at 50 amps or until homogenous. RNA was extracted and purified according to manufacturer (Molecular Research Center Inc.) instructions for total RNA isolation.

### 2.4. cDNA Synthesis

cDNA was generated from 1 µg of total RNA using BioRad iSCRIPT cDNA Synthesis Kit according to the manufacturer’s instructions.

### 2.5. Quantitative Real Time PCR

Quantitative Real Time PCR (qRT-PCR) was used to determine the relative quantities of *SULT* transcribed in different species of schistosomes. 150 µg of cDNA was used for relative quantification with gene-specific primers and iTaq™ Universal SYBR^®^ Green Supermix (BioRad, Hercules, CA, USA) containing hot-start iTaq DNA polymerase, dNTPs, MgCl2, SYBR^®^ Green I dye, and ROX reference dye. Primers were designed using PerlPrimer v1.21 (Supplementary Table S1) for each *SULT* gene (*SmSULT*: Smp_089320; *ShSULT*: Sha_104171; *SjSULT*: FN317462.1) and assayed for efficiency at 1:0, 1:5, 1:25, and 1:100 cDNA concentrations.

*Gapdh*, β-*Tubulin*, and *Actin* were used as endogenous controls for each species. The qRT-PCR reaction was performed in 10 μL reaction and contained 5µL iTaq Universal SYBR^®^ Green Supermix (BioRad), 3 µL cDNA (50 µg/µL), 1 µL each of forward and reverse primers 100 µM. The qRT-PCR profile was 50 °C for 2 min, 95 °C for 10 min, 40 cycles of 95 °C for 15 s, 60 °C for 1 min, and a final step of 60 °C for 5 min (Applied Biosystems 7500 FastReal-Time PCR System, Waltham, MA, USA).

Comparative sulfotransferase expression between schistosome species was calculated based on qRT-PCR amplification results, using an equation to adjust according to an internal reference primer and primer amplification efficiency (Supplementary Figure S1). This approach is algebraically equivalent to the ∆∆Ct method.

### 2.6. Digital PCR

Digital PCR was also used to measure the absolute concentration of *SULT* transcripts in total RNA. cDNA was prepared as previously described. The Bioanalytics and Single Cell Core at UTHSCSA were given cDNA at 50 ng/µL and primers at 100 µM. The sample was diluted to 20 ng/µL (“1×” concentration) and the primers were diluted to 50 µM. Digital PCR using Gene Expression Assay (Fluidigm, South San Francisco, CA, USA) was performed on a BioMark HD (Fluidigm) using EvaGreen (Biotium, Fremont, CA, USA) DNA-intercalating fluorescent dye as a reporter. The ddPCR primer sequences are in Supplemental Table S2.

### 2.7. Tritiated Drug Labeling

Tritiated OXA was synthesized by the Center for Innovative Drug Discovery (CIDD) using radiolabeled NaBH_4_ (Moravek Biochemicals, Brea, CA, USA) at 100 mCi. For each mole of OXA in the aldehyde form, a single Ci was added and reacted at room temperature to completion and detected by thin-layer chromatography. The levels of radioactivity were determined by blotting 10 µL of the final product onto filter paper which were then counted via liquid scintillation counter (Beckman LS 6500 Scintillation Counter, Port Jefferson, NY, USA) for 10 min for each reaction.

### 2.8. OXA Activation Assay

The ability of a recombinant protein to activate OXA was tested by quantifying how much tritiated OXA was able to bind DNA. For each reaction, 100 μCi of [3H]OXA was solubilized in 2 µL DMSO and added to 10 µL of a 3′-phosphoadenosine-5′-phosphosulfate (PAPS) mix containing ATP and MgCl_2_ at 50 mM each, and PAPS at 1 mM [6,29]. The radiolabeled OXA and PAPS mix was then added to 90 µL of recombinant protein with 10 ng/µL sheared *S. mansoni* (gDNA) as a final target for activated [3H]OXA. The mixture was incubated at 37 °C for 2.5 h when testing worm extract. Then the reaction was stopped with 3 volumes of 1 mM sodium bicarbonate containing 0.1% SDS (*w*/*v*). Afterward, the reaction was extracted 3 times using 2 volumes of dichloromethane. A 10 µL aliquot of the aqueous phase was collected onto a small square of filter paper in a scintillation vial and then counted via a liquid scintillation counter (Beckman LS 6500 Scintillation Counter) for 10 min for each reaction.

### 2.9. RNAi

First, primers amplifying a 192–592 bp section of the gene coding region were generated using the PrimerDesign tool by IDTdna (Supplementary Table S3). PCR was performed to produce the amplified gene section, followed by confirmation of amplification by running the PCR product on a 1% agarose gel. T7 promoters were then added to both the forward and reverse primer to flank the PCR product and confirmation of amplification was performed via 1% agarose gel. The PCR product with T7 promoters was then used as the template for in vitro transcription of the dsRNA. The dsRNA was placed in a 37 °C water bath, 4 h to overnight, then treated with DNAse to remove contaminants. Ammonium acetate (3 M) was added, followed by 100% ethanol to precipitate the RNA. The RNA was left at this step for 2 h to overnight, depending on the yield of RNA. The sample was then centrifuged at 14,000 rpm, forming an RNA pellet. The pellet was washed twice with ethanol. On the last wash, the supernatant was removed, and the ethanol was allowed to evaporate. The pellet was then resuspended in nuclease-free water. The concentration of RNA was then determined using the Thermo Scientific NanoDroprop 1000 spectrophotometer.

Adult male schistosomes were treated with 30 µg/mL dsRNA of either *S. mansoni* *SULT*, *S. haematobium* *SULT*, or *S. japonicum* *SULT* or irrelevant control (*Drosophila* Nautilis gene) RNAs on day 0, 3, 7 and 11. The worms were treated with 143 µM CIDD-0066790 on day 6. CIDD-0066790 is capable of killing 85–100% *S. mansoni*, 40% *S. haematobium,* and 83% *S. japonicum* [26]. The worms were observed for 14 days, pooled, and quick-frozen. Observation included notes on worm health, viability; lack of motility, shedding of tegument, blebbing of tegument, internal vacuolization, lethargy, and being opaque.

There were three biological replicates, 10 male worms per replicate that were pooled to conduct qPCR due to the limited number of worms. This silencing experiment was done twice to confirm gene expression ablation (pilot + full experiment). Treatment with the drug was performed once in triplicate. The qPCR samples were performed in triplicate to account for the variation of the run (technical replicates).

### 2.10. Whole Worm In Situ Hybridization

This protocol was adapted from Collins et al. [30,31].

To fix worms, freshly collected *S. mansoni*, *S. haematobium*, or *S. japonicum* parasites were placed in a 15 mL conical tube with 10 mL DMEM + 10% FEB. 1/10th volume of a 2.5% anesthetic ethyl 3-aminobenzoate methanesulfonate (Sigma-Aldrich, St. Louis, MO, USA) was used to separate paired worms for 10 min on a rocker at room temperature. The parasites were then killed with 1 mL of 0.6 M MgCl_2_ for 1 min. MgCl_2_ solution was then replaced with 10 mL 4% Formaldehyde in Phosphate Buffered Saline with 0.3% Triton x-100 (PBSTx) for 4 h on a rocker at room temperature to fix the worms. After 4 h of incubation, the worms were then rinsed with 10 mL 1× PBSTx for 10 min. Worms were then dehydrated in methanol and kept at −20 °C until used. Dehydrated worms were rehydrated with 10 mL 50% methanol in 1× PBSTx for 10 min and then incubated for 10 min in 10 mL 1× PBSTx at room temperature on a rocker. Worms were bleached in a solution [9ml H_2_O, 500 μL formamide, 250 μL 20× SSC (3M sodium chloride, and 3M sodium citrate, pH7.0), 400 μL 30% H_2_O_2_], and incubated for 1 hr at room temperature under bright light. Worms were rinsed twice with 1× PBSTx for 10 min for each wash. Worms were then incubated with 5 μg/mL Proteinase K (Invitrogen, Carlsbad, CA, USA) in 1× PBSTx for 45 min at room temperature. Worms were post-fixed in 10 mL of 4% Formaldehyde in 1× PBSTx for 10 min at room temperature. Then, worms were washed in a 1:1 ratio of PBSTx and pre-hybridization solution for 10 min at room temperature. Male parasites (5–6) were placed into 48-well-sized baskets (Intavis Bioanalytical Instruments) and incubated in pre-hybridization solution (50% deionized formamide, 5× SSC, and 1.2% H_2_O_2_) for 2 h at 52 °C. The worms were then incubated in 300 μL of hybridization solution (prehybridization solution with 10% dextran sulfate) and 150 ng/mL of Riboprobe generated for *SULT* RNA overnight at 52 °C (Supplemental Table S3). The worms were then washed twice, for 30 min each, in wash hybridization buffer, 2× SSC + 0.1% Triton-X, and lastly with 0.2× SSC + 0.1% Triton-X. The worms were then washed twice in 300 μL TNT (0.1M Tris.HCl (pH 7.5), 0.15M NaCl, 0.05% Tween^®^-20) for 10 min each.

### 2.11. Oxamniquine Localization

To visualize the localization of activated OXA in *S. mansoni* adult worms, [3H]OXA was incubated with living adult worms in a method similar to OXA-derivative drug screening [32]. [3H]OXA suspended in 100% dichloromethane was allowed to evaporate and the resulting pellet weighed. The [3H]OXA pellet was solubilized in 100% DMSO to a concentration of 50 mM. [3H]OXA was administered to the adult male worms 2–24 h after harvesting from the hamsters. It was administered to the worms in media at a final concentration of 143 µM. [3H]OXA was incubated with the worms at 37 °C, 5% CO_2_ for 45 min, then the media was removed without disturbing the worms. The worms were washed with plain media 3× to remove any unincorporated [3H]OXA. Worms were incubated in media for a period of 10–14 days with groups of 10 worms being removed and fixed in 4% paraformaldehyde every 2 days.

Fixed worms were embedded in paraffin, sectioned by the UTHSCSA Histology and Immunohistochemistry core facility to a thickness of 7 µm, and mounted on glass slides. The sections were de-paraffinized to water and pretreated with Photo-Flo (Kodak Professional Photo-Flo 200) for 10 min. Under dark room conditions, emulsion (Kodak NTB Emulsion) was melted in a 42 °C water bath and diluted 1:2 in distilled water. The slides were dipped into the emulsion in pairs (sample side out) and hung on a line for 4 h or until dry to the touch. The slides were packed into slide boxes containing desiccant and wrapped three times in tape and foil to prevent light exposure. The slides were incubated at 4 °C in the dark for 6 weeks. Slides were recovered from the boxes in a dark room and developed (Kodak D-19 developer) for 1 min. Slides were then washed in Millipore water for 1 min and fixed in Kodak fixative for 1 min.

Developed slides were then lightly H&E stained, mounted by the UTHSCSA Histology and Immunohistochemistry core facility, and photographed in the UTHSCSA Core Optical Imaging Facility at 100× to identify silver grain placement in the adult worms using a Nikon N-Storm Super Resolution Microscope equipped with Elipse Ti inverted TIRF microscope, and two high resolution CCD cameras: Photometrics Cool SNAP HQ2 and Andor iXon3 EMCCD.

### 2.12. In Silico Analysis

The following public domain tools were used for sequence and transcriptome analyses: NCBI-BLAST 166 (http://blast.ncbi.nlm.nih.gov/Blast.cgi, accessed on 15 December 2020), PerlPrimer v1.21, http://schisto.xyz/, accessed on 15 December 2020 for genome version 5 and 7. Transcriptome information was obtained from genome version 5 and 7 (http://schisto.xyz/, accessed on 15 December 2020) [33], and SchistoCyte Atlas (http://www.collinslab.org/schistocyte, accessed on 15 December 2020) [34].

### 2.13. Statistics

Statistical analysis for digital PCR studies was performed using GraphPad Prism software. One-way analysis of variance (ANOVA) was performed to compare absolute values of three individual experiments. Statistical analysis for the Kaplan-Meier curves was performed using R software scripts [35].

## 3. Results

### 3.1. Activation Assay to Determine Mode of Action of CIDD-0066790

**Figure 1:**
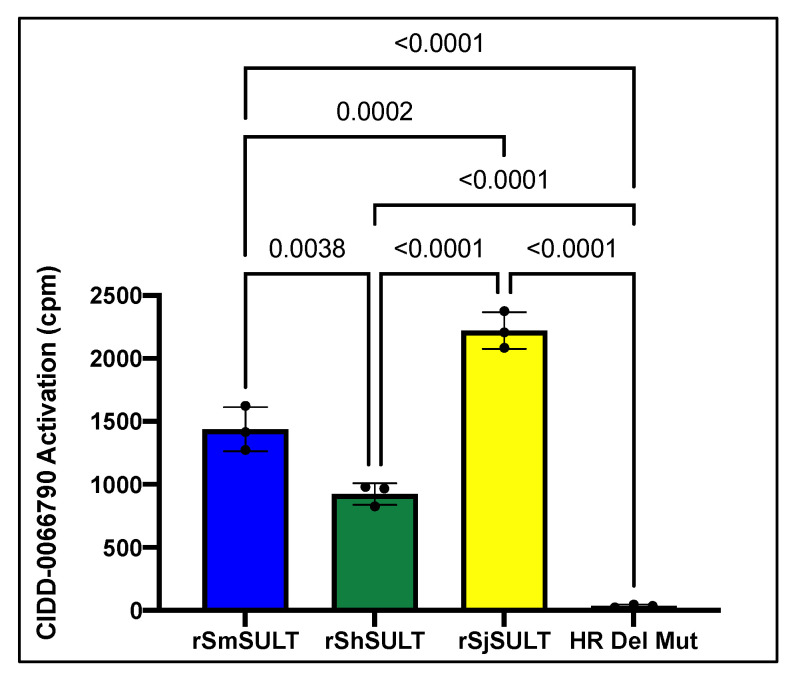
*S. mansoni*, *S. haematobium*, and *S. japonicum* Recombinant Protein CIDD-0066790 Activation Assay. The Y-axis represents scintillation counts per minute (cpm). Higher sulfotransferase activity is indicated by higher cpm. *rSmSULT* is a known sulfotransferase and is acting as a positive control. rHRdelMut is a known defunct sulfotransferase and is acting as a negative control. Background levels of radiation were subtracted from all reactions prior to graphing. Error bars depict standard deviation from three replicates.

We used radiolabeled CIDD-0066790 in an activation assay to demonstrate that the recombinant *SULTs* from all three human schistosome species could sulfate CIDD-0066790 compared to a control (Figure 1).

### 3.2. RNAi Knockdown of SmSULT to Confirm Mode of Action of CIDD-0066790

**Figure 2:**
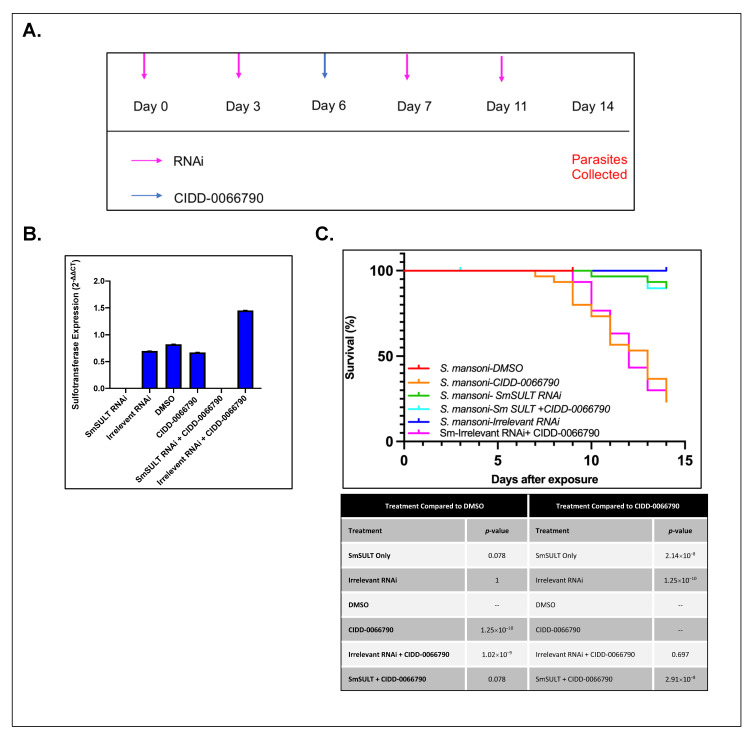
Silencing of *S. mansoni* sulfotransferase results in resistant parasites upon challenge with CIDD-0066790. (**A**) RNAi treatment schematic. Adult male parasites were sorted, 10 per well, on day 0 and subsequently treated with 30 μg/mL of RNAi per mL of media. RNAi was supplemented every other day, as highlighted with pink arrows. On day 6, parasites were treated with CIDD-0066790 (blue arrow) at a final concentration of 143 µM. Parasites were incubated with drug for 45 min. After 45 min, parasites were washed 3 times with DMEM + 10% FBS to wash any residual compound. After 14 days, the parasites were collected and quick frozen for RNA isolation. (**B**) *SmSULT*-specific knockdown using RNA interference. Quantitative PCR (qRT-PCR) was conducted to confirm gene silencing of *S.* *mansoni* sulfotransferase (*SmSULT*). Gene expression in *SmSULT* RNAi group was significantly reduced by about 99% when compared to other treatment groups. *GAPDH* was used as the endogenous control. There was a no-treatment group that was just male worms (not shown), which was used as the control, which was the sample that was set to 1. (**C**) Survival curve analysis of adult male *S. mansoni* parasites exposed to CIDD-0066790. Irrelevant RNAi, *SmSULT* RNAi alone, and *SmSULT* RNAi + CIDD-0066790 had 90%+ survival and were displaying healthy characteristics (no signs of blebbing, and internal vacuolization) and movement.

To further demonstrate that the schistosome sulfotransferases were responsible for activating the OXA derivatives. RNAi was performed for all three human *Schistosoma* sulfotransferases. Adult male parasites were used due to their increased susceptibility to therapies such as OXA and its derivatives [32]. The parasites were incubated with 30 μg/mL dsRNA on day 0 and day 3 and treated with CIDD-0066790 on day 6 (Figure 2A). On day 7 the parasites were incubated with dsRNA again. Media and RNAi were administered every other day for the remainder of the treatment. The parasites were constantly exposed to RNAi throughout treatment to ensure efficient gene silencing.

In these studies, parasites were treated with either DMSO (no treatment), an irrelevant RNAi, *SULT* RNAi, CIDD-0066790, irrelevant RNAi + CIDD-0066790, or *SULT* RNAi + CIDD-0066790. To ensure the gene was silenced efficiently, quantitative PCR (qPCR) was performed for all groups. Gene expression analysis showed a significant reduction in *SmSULT* expression in both the *SULT* RNAi and the *SULT* RNAi + CIDD-0066790, with 99% knockdown efficiency (Figure 2B). There was a slight upregulation in *SmSULT* expression seen in the irrelevant RNAi + CIDD-0066790 group, which was consistent with both pilot and replicate experiments. However, all other treatment groups showed expected expression levels. *S. mansoni* parasites treated with irrelevant RNAi, *SULT* RNAi, and DMSO resulted in 90% survival. *S. mansoni* parasites treated with CIDD-0066790 and irrelevant RNAi + CIDD-0066790 resulted in 15–20% survival, while parasites treated with *SULT* RNAi + CIDD-0066790 resulted in 90% survival (Figure 2C).

**Figure 3:**
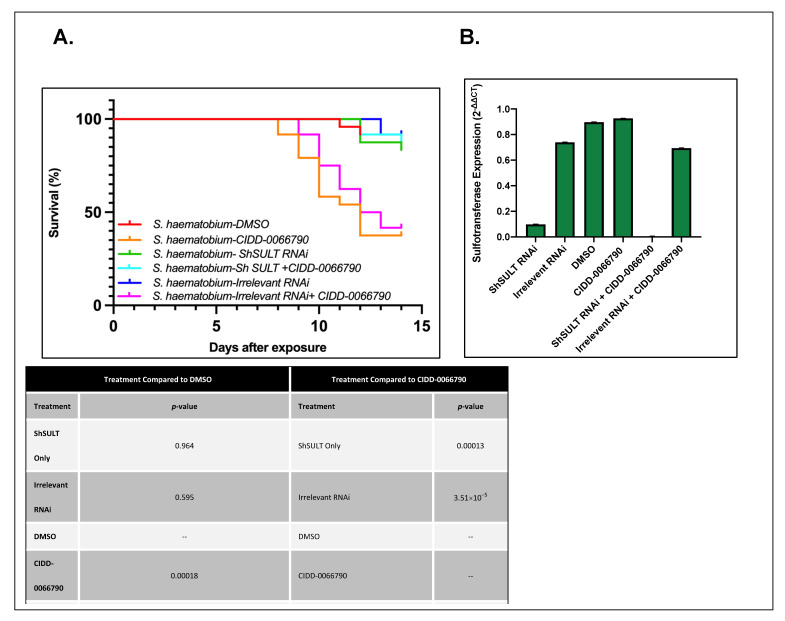
Silencing of *ShSULT* results in resistant parasites upon challenge with CIDD-0066790. (**A**) Kaplan-Meier survival curve of *S. haematobium* parasites. Survival curve analysis of adult, male *S. haematobium* parasites challenged with CIDD-0066790 with or without RNAi treatment. Parasites were cultured for 14 days. RNAi treatment occurred as stated in schematic above of Figure 1A. Irrelevant RNAi and *ShSULT*. RNAi alone had about 90%+ survival and displayed healthy characteristics (no signs of shedding, blebbing, and interval vacuolization) and movement confirming that *ShSULT* is required for CIDD-0066790 activation. (**B**) *ShSULT*-specific knockdown using RNA interference. Quantitative PCR (qPCR) was conducted to confirm gene silencing of *S. haematobium* sulfotransferase (*ShSULT*). Gene expression in *ShSULT* RNAi group was significantly reduced by about 95–99% when compared to other treatment groups. *GAPDH* was used as the endogenous control. There was a no-treatment group that was just male worms (not shown), which was used as the control, which was the sample that was set to 1.

*S. haematobium* demonstrated a similar result to *S. mansoni* in that knockdown of *ShSULT* showed a significant reduction in *ShSULT* expression in both the *ShSULT* RNAi and the *ShSULT* RNAi + CIDD-0066790 treatment groups, with 95–99% knockdown efficiency (Figure 3B). All other groups expressed similar, expected levels of *ShSULT*. *S. haematobium* parasites treated with irrelevant RNAi, *ShSULT* RNAi, and DMSO resulted in 88% survival. *S. haematobium* parasites treated with *ShSULT* RNAi + CIDD-0066790 resulted in 90% survival showing no signs of blebbing and internal vacuolization and parasite movement, confirming that *Sh*SULT is required to activate CIDD-0066790. Irrelevant RNAi + CIDD-0066790 resulted in 40–50% survival, as expected (Figure 3A).

**Figure 4:**
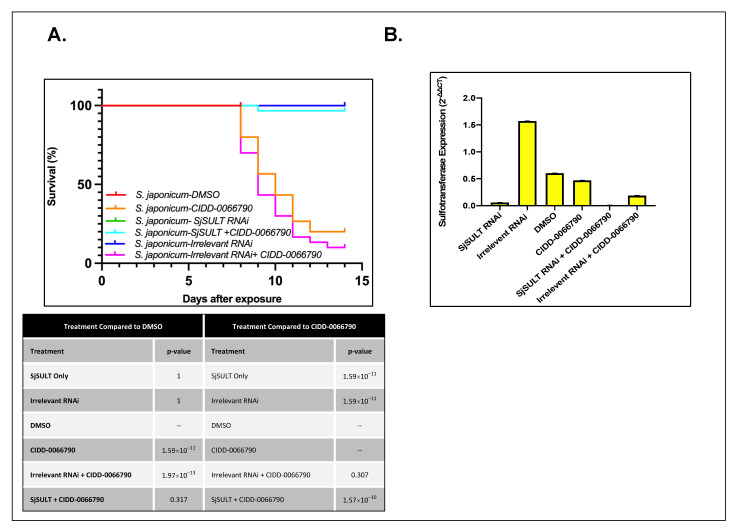
Silencing of *SjSULT* results in resistant parasites upon challenge with CIDD-0066790. (**A**) Kaplan-Meier survival curve of *S. japonicum* parasites. Survival curve analysis of adult, male *S. japonicum* parasites challenged with CIDD-0066790 with or without RNAi treatment. Parasites were cultured for 14 days. RNAi treatment occurred as stated in schematic of Figure 1A. Irrelevant RNAi and *SjSULT* RNAi alone had 90%+ survival and were displaying healthy characteristics (no signs of shedding, blebbing, and interval vacuolization) and movement confirming that *SjSULT* is required for CIDD-0066790 activation. (**B**) *SjSULT*-specific knockdown using RNA interference Quantitative PCR (qPCR) was conducted to confirm gene silencing of *S. japonicum* sulfotransferase (*SjSULT*). Gene expression in *SjSULT* RNAi group was significantly reduced by about 95–99% when compared to other treatment groups. *GAPDH* was used as the endogenous control. There was a no-treatment group that was just male worms (not shown), which was used as the control, which was the sample that was set to 1.

*S. japonicum*, showed a similar result as *S. mansoni* and *S. haematobium*. Gene expression analysis displayed a reduction in *SjSULT* expression in the *SjSULT* RNAi and the *SjSULT* RNAi + CIDD-0066790 groups when compared to controls (Figure 4B). *SjSULT* expression in the remaining treatment groups was similar to control groups. *S. japonicum* parasites treated with irrelevant RNAi, *SjSULT* RNAi, and DMSO resulted in 100% survival. *S. japonicum* parasites treated with CIDD-0066790 alone and irrelevant RNAi + CIDD-0066790 resulted in 26% and 13% survival, respectively, while parasites treated with *SjSULT* RNAi + CIDD-0066790 resulted in 96% survival (Figure 4A).

### 3.3. SmSULT Transcription in Life Stages

**Figure 5:**
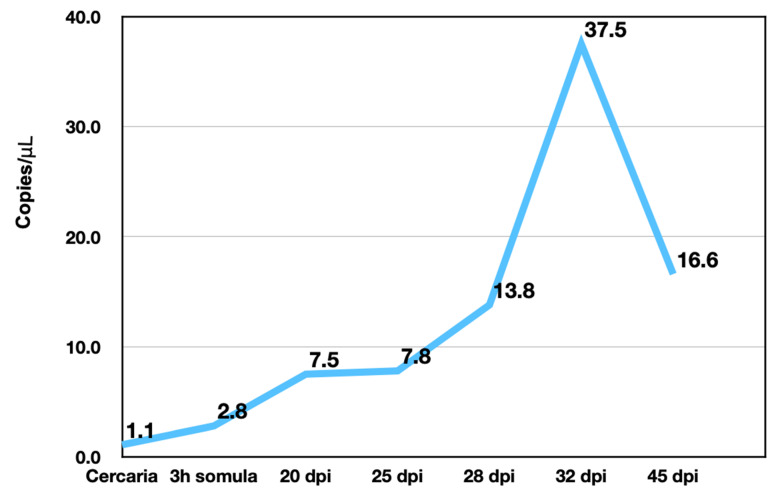
Concentration (copies/μL) of *SmSULT* expressed in male infections throughout the life stages.

Digital PCR (ddPCR) was used to quantify *SmSULT* expressed in male schistosomes at cercariae, 3 hr schistosomula, day 20 immature worms, day 25 immature worms, day 28 immature worms, day 32 worms, and day 45 adult worms (Figure 5). The rise in *SULT* expression started at 3hr post-infection (pi) with the transformation of cercariae to schistosomulae and peaks at 32 days post-infection (dpi). The question of whether the copies of *SULT*/μL correlated with the ability of OXA to kill the life stage was addressed.

**Figure 6:**
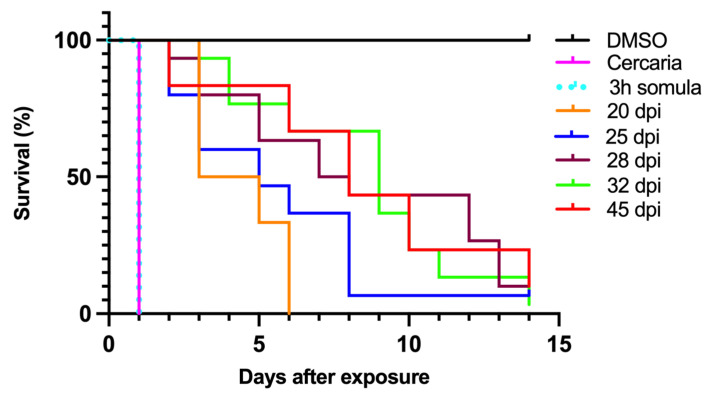
OXA treatment of life stages of *S. mansoni*.

Figure 6 shows OXA can kill cercariae and 3hr schistosomula in 24 h, 20-day pi in 6 days, 25 dpi 95% in 8 dpi and 14 dpi to kill 100% of day 28, 32, and 45 dpi worms.

**Figure 7:**
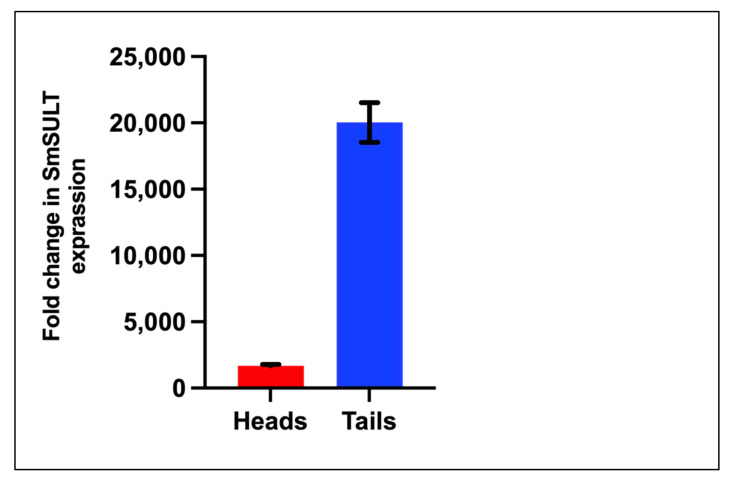
Comparison of *SmSULT* in cercarial tails vs. heads using qRT-PCR.

The data in Figure 7 show a higher level of *SmSULT* in cercarial tails than heads.

### 3.4. Cross-Species SULT Transcripts

**Figure 8:**
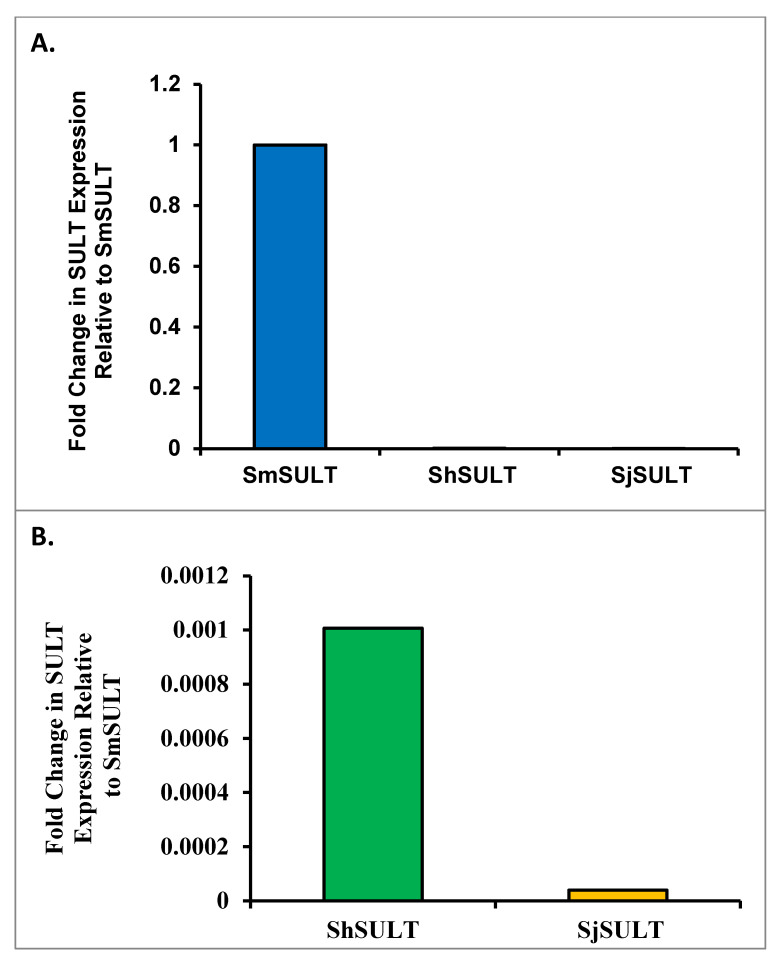
*SULT* Expression Cross-species Comparison qRT-PCR. Transcripts of the *SULT* genes from *S. mansoni*, *S. haematobium*, and *S. japonicum*, were evaluated by quantitative reverse transcriptase PCR. Species-specific primers were designed and validated. Relative quantities were determined by an algebraic equivalent to the ∆∆Ct method which allowed adjustment for differences in primer amplification efficiency. (**A**) Transcript levels in all three species relative to *S. mansoni*. (**B**) Transcript level comparisons between *S. haematobium* and *S. japonicum*, with *S. mansoni* omitted to more clearly show the difference between *ShSULT* and *SjSULT*.

PCR was used to evaluate *SULT* transcript levels of *S. mansoni* adult worms compared to the adult worms of *S. haematobium* and *S. japonicum*. The results were calculated with a formula to allow cross-species comparison (Supplementary Figure S1). The results indicate *SULT* expression in *S. haematobium* is nearly 100× less than *S. mansoni*. *S. japonicum* *SULT* transcripts are 10× below *S. haematobium* (Figure 8).

### 3.5. SULT Transcripts: S. mansoni vs. S. haematobium vs. S. japonicum ddPCR

**Figure 9:**
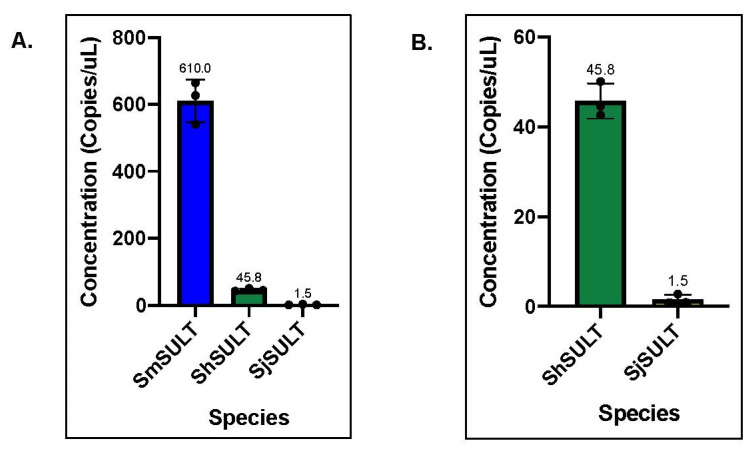
Comparison of concentrations (copies/μL) of sulfotransferase transcripts across all three *Schistosoma* species. (**A**) Mean concentration of *SmSULT* (610 copies/μL), *ShSULT* (45.77 copies/μL), and *SjSULT* (1.533 copies/μL) per well. (**B**) Concentration of *ShSULT* and *SjSULT* for closer comparison. Based on 3 individual experiments. Std. Deviation: *Sm* = 63.53, *Sh* = 3.48; *Sj* = 1.097.

Droplet digital PCR (ddPCR) was used to obtain absolute quantities of *SULT* transcripts, rather than relative quantities, in *S. mansoni* mature adult worms compared to *S. haematobium* and *S. japonicum* mature adult worms. Concentrations of sulfotransferase enzyme expression were obtained for all three species; *S. mansoni* (610 copies/μL), *S. haematobium* (45.8 copies/μL), and *S. japonicum* (1.5 copies/μL) (Figure 9A). The difference between *S. haematobium* and *S. japonicum* is further highlighted in Figure 9B. Based on these data, there is about a 13-fold difference in expression between *S. mansoni* and *S. haematobium* (Figure 9A), and a 30-fold difference in expression between *S. haematobium* and *S. japonicum* (Figure 9B). To stress the difference in expression between *S. mansoni* and *S. japonicum*, there is over a 400-fold difference between these two species (Figure 9A).

### 3.6. Whole Organism Localization of Schistosoma Sulfotransferases

**Figure 10:**
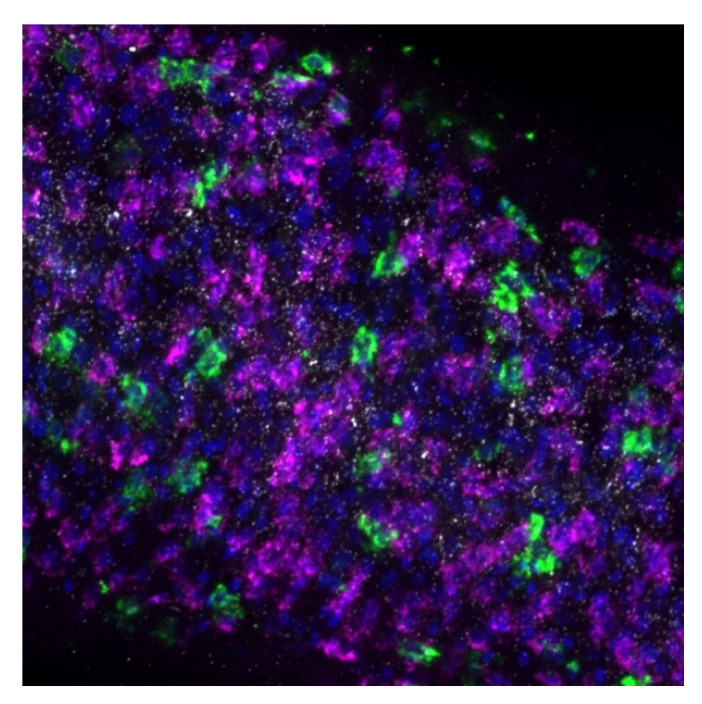
Fluorescence in situ hybridization of *SmSULT*. Body section of male worm showing *SmSULT* distribution. Cells positive for *SmSULT* stained positive for TAMRA-Tyramide, magenta. DAPI (blue) was used at a final concentration of 1 ug/mL to stain cell nuclei. FITC-positive cells are *Schistosoma* stem cells, positive for *Schistosoma histone H2B* [30,31]. *SmSULT* and *H2B* probes were used at a final concentration of 150 ng/mL. FISH was performed on 4–5 whole worms.

**Figure 11:**
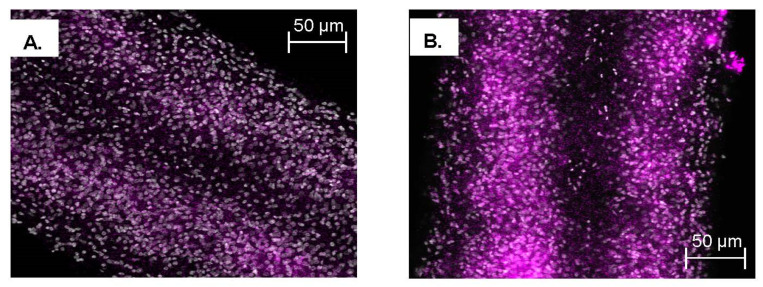
Fluorescence in situ hybridization of *ShSULT*. Midsection of *S. haematobium* male. Cells positive for *ShSULT* stained positive for TAMRA-Tyramide, magenta. *ShSULT*-specific probe was used at a concentration of 100 ng/mL DAPI was not included. FISH was performed on whole worms. (**A**) Z-stack image. (**B**) Whole section. These photos are representative of photos from four worms.

We used fluorescence in situ hybridization (FISH) to visualize the differences in expression between species using whole worms. Images captured from whole worm fluorescence in situ hybridization of adult male parasites suggest that the sulfotransferase is expressed throughout the parasite in a variety of cell types, in great abundance in *S. mansoni* (Figure 10 and Figure S1, Video S1). The same process was used for *S. haematobium* with cells throughout the entire body expressing *ShSULT* (Figure 11). After a number of attempts, we were not successful in obtaining the whole worm in situ hybridization results for *S. japonicum* even though the positive controls demonstrated that the process was working. We hypothesize that this was due to the low amount of transcript present in *S. japonicum*.

### 3.7. [3H]OXA Localization

**Figure 12:**
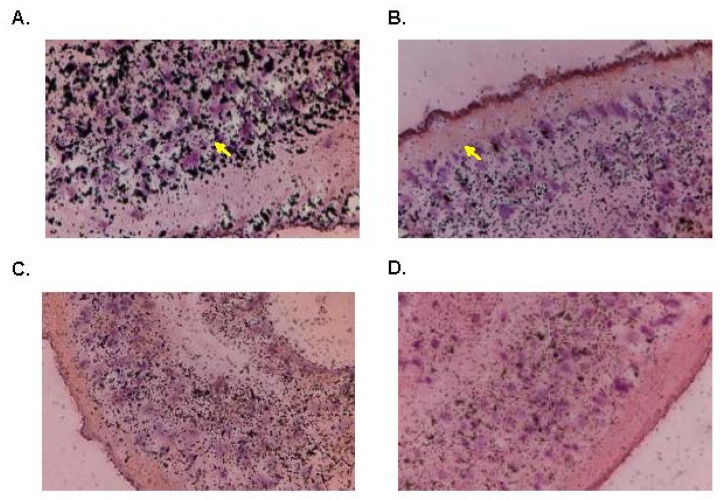
Silver Grain Placement in Radiolabeled-OXA Treated Worms. Silver grains in H&Estained parasite sections. Yellow arrows indicate prominent clusters of silver grains. The clustering is less prominent the longer the worm is exposed to OXA. (**A**) Day 2, (**B**) Day 4, (**C**) Day 6, (**D**) Day 12.

In order to identify where activated OXA bound within the schistosome after activation, male worms were treated with [3H]OXA. Sections of treated worms showed the localization of the labeled OXA over a period of 12 days. Silver grains localize to the radiolabeled OXA on the treated slides. The results indicate an initial concentration of silver grains in the tegument, gut lining, and parenchymal cells. The silver grains become more widely dispersed over 12 days (Figure 12).

## 4. Discussion

The three major species, that cause human schistosomiasis, *S. mansoni*, *S. haematobium*, and *S. japonicum*, account for over 99.5% of all global cases of the disease [36]. Currently, there is only one method of treatment, a single dose of PZQ. In 2013, the mechanism of action was elucidated for another schistosomicidal pharmaceutical, OXA. The data produced by Valentim et al. [21] allowed for questions to be asked; why is OXA only effective at certain life stages of the parasite? Why is OXA effective in *S. mansoni* but not in the other major schistosome species? Is the mode of action the same for all the OXA derivatives, that is, sulfation by a sulfotransferase, as described by Valentim et al. [21] and Guzman et al. [37]? Where and to what extent is the *SULT* expressed in the adult parasite and does this account for the mechanism of killing? We used CIDD-0066790 for these studies as it will kill all three human species of *Schistosoma* [25].

One possible explanation for resistance to OXA by *S. haematobium* and *S. japonicum*, respectively, is that the activating *SULT* enzyme is differentially expressed across the three species. This is a possibility given that the differences in amino acid residues in the *SULT* active site across species do not seem to be responsible for differences in OXA schistosomicidal activity [29].

Whole worm transcript levels of the sulfotransferase in *S. haematobium* and *S. japonicum* indicate the little-to-no presence of *SULT*, which may provide a partial explanation as to why *S. haematobium* and *S. japonicum* are resistant to OXA [29]. Regardless of these differences in expression, the designed OXA derivative, CIDD-0066790, could be activated by recombinant *SULT* from each schistosome species (Figure 1). As the activating agent of OXA is the sulfotransferase, and the derivative studied is based on OXA, we hypothesized the sulfotransferase is also the activating enzyme of the derivatives. To demonstrate this, the sulfotransferase was silenced in each of the schistosome species and challenged with CIDD-0066790. This approach would determine if the OXA derivative would kill the three schistosome species, indicating a change in the mode of action or if the OXA mode of action was retained. Using RNAi, silencing of the sulfotransferase in each species resulted in resistance to the OXA derivative and reinforced the notion that the mechanism of killing was the result of sulfation of CIDD-0066790 (Figure 2, Figure 3 and Figure 4). Evidence to date suggests that the ability of OXA, or its derivatives, to fit into the binding pocket and accept the transfer of a sulfur group from a sulfur donor is more likely the reason for activation and subsequent killing ability [24,25,29].

It was previously proposed that differences in expression was the underlying cause of OXA ineffectiveness against the other two *Schistosoma* species [21]. Therefore, we aimed to determine expression patterns between species using qPCR and highly sensitive, droplet digital PCR (ddPCR). We compared transcript levels of *SULT* across the three major species using qPCR. Normally, a cross-species comparison is not ideal due to the lack of appropriate internal references. Using multiple homologous reference genes as a basis for comparison, a formula was developed to compare schistosome *SULTs* across species (Supplemental Figure S1). The initial qPCR results showed far less *SULT* transcript in *S. haematobium* and *S. japonicum* compared *to S. mansoni* (Figure 8). This relative transcript level was independently confirmed using droplet digital PCR (ddPCR), which allowed for the absolute quantification of *SULT* transcripts. Based on the ddPCR data, there is about a 13-fold difference in expression between *S. mansoni* and *S. haematobium*, and a 30-fold difference in expression between *S. haematobium* and *S. japonicum*. To stress the difference in expression between *S. mansoni* and *S. japonicum*, there is over a 400-fold difference between these two groups (Figure 9). As OXA is a prodrug that requires activation via a *SULT*, there is a correlation between the failure of OXA to kill the other two human schistosome species and the low amounts of *SULT* in *S. haematobium* and *S. japonicum*. This disparity could account for OXA’s failure to kill even if the SULTs are able to bind OXA with similar affinities [37] and activate OXA in an in vitro setting [23].

CIDD-0066790 is effective against all three species with efficacy ranging from 40–85% depending on the schistosome species, thus the hypothesis that differences in sulfotransferase expression accounts for the difference in killing needs further exploration. While qPCR and ddPCR were informative, verification of these results is necessary at the protein level. The development of an antibody that recognizes endogenous *SULT* protein is a critical priority that has not yet been achieved. Polyclonal and monoclonal antibodies that recognize recombinant, but not native protein, have been produced (unpublished data). The development of an antibody that can recognize native protein would allow for further characterization of *SULT* protein expression in all different worm species as well as life stages.

*S. mansoni* is sensitive to OXA, but only in certain life stages such as the mature adult phase. Transcript levels of *SmSULT* across several life stages, including cercariae, schistosomulae, immature worms (Day 20, 25, and 28), and mature adult worms correlate with OXA and OXA derivative sensitivity. Previous studies demonstrated a stage-specific susceptibility of *S. mansoni* to OXA treatment [12,38]. Now that the mechanism of action of OXA has been elucidated [21,25], the expression of *SULT* can be correlated with OXA killing (Figure 5 and Figure 6). Interestingly, at the infectious cercariae stage, the cercarial tail expresses higher levels of *SmSULT* than the cercarial head. This may suggest a biological role for the sulfotransferase in the cercarial tail. Recent studies have examined the differences in transcription and translation between cercarial heads and tails [39]. Transcriptome data is consistent with the high level of expression in cercariae [40]. Furthermore, sex-associated differences in susceptibility to OXA treatment have been noted as adult male worms are more susceptible to treatment. Transcript level comparison between male and female adult worms also correlates with that susceptibility [32].

To address the lingering question of where the sulfotransferase is expressed in adult worms, fluorescence in situ hybridization was used to determine expression localization. The sulfotransferase appeared to localize throughout the worm in *S. mansoni* and *S. haematobium*, where it appeared to localize to the parenchyma and outer tegumental region. We assume the same is true for *S. japonicum*. However, the low level of *SjSULT* expression was likely the reason we were not able to obtain in situ results for this species. Regarding the identification of specific cell types, the schistosome field is just starting to uncover cell-specific markers [40]. However, transcriptome data from genome version 5 and 7 and the SchistoCyte Atlas identify *SmSULT* (Smp_089320) as expressed in multiple cell types in mature and immature male and female *S. mansoni* [33,34]. At 24 hrs post cercarial transformation to schistosomulae, *SmSULT* expression begins to rise in male schistosomulae. Expression increases through days 21, and 28 and peaks at day 35. In females according to [35,36] expression begins to increase at day 21, peaks at day 28, and then declines, consistent with our results for male schistosomes (Figure 5).

In conclusion, our data demonstrate that many cells in this multicellular eukaryotic pathogen express *SULT*, and this level of expression can promote the activation, and thereby the killing ability of OXA and its derivatives.

In order to further investigate and confirm the anatomical tissue responsible for OXA’s anthelminthic effect, we used silver grain localization to locate adduct formation with radioactive OXA. Pica-Mattoccia et al. [24] demonstrated that OXA formed adducts with DNA, so we wanted to identify where in the worm these adducts were forming. Initial results showed a concentration of [3H]OXA along the gut lining, tegument, and subtegumental and parenchymal cells. Throughout a 12-day period, these concentrations slowly dispersed evenly throughout the tissue which correlates with the time it takes OXA (14 days) to kill adult worms in vitro. The findings presented in this article are highly significant, as they provide further insight into the mechanism of action of OXA and the OXA derivatives. As mentioned earlier, the proposed mechanism of action of these derivatives is that they are prodrugs activated by the sulfotransferase enzyme. One of two reactions is proposed to occur: (1) decay of the sulfate forms a reactive electrophilic product and subsequent alkylation of DNA, proteins, and other macromolecules leads to the death of the parasite due to the disruption of diverse cellular and metabolic functions [21,24], and (2) experimental evidence suggests the sulfuric acid monoester directly reacts with cellular nucleophiles in an S_N_2-like reaction with sulfate as a leaving group, which disrupts cellular and metabolic functions leading to death [23]. By visualizing sulfotransferase expression, we suggest that such widespread expression of the enzyme contributes to the killing capacity of the derivatives in a eukaryotic multicellular parasite.

The recently elucidated mechanism of action for the parent drug, OXA, has created an exploitable opening for directed drug development [23,25,32]. By understanding the mechanism by which the major species of schistosomes are susceptible to OXA-derived compounds, pharmaceuticals can be designed to complement praziquantel, the drug of choice to circumvent praziquantel resistance and improve its efficacy.

## Data Availability

Data is contained within the article or Supplementary material. See Section 2.12 for public domain data.

## Supplementary Materials

The following supporting information can be downloaded at: https://www.mdpi.com/article/10.3390/pharmaceutics14071416/s1, Figure S1. Formula for Cross Species *SULT* Transcript Comparison. Table S1. Quantitative PCR Primer Sequences. Table S2. Digital PCR Primer Sets. Table S3: *SULT* T7 Primer Sequences. Video S1: In situ hybridization of *SmSULT* in adult *S. mansoni* head.
