# Supplementary material for: Schistosome Sulfotransferases: Mode of Action, Expression and Localization
Source: Pharmaceutics. 2022 Jul 6;14(7):1416. doi: 10.3390/pharmaceutics14071416 (PMC9323829; doi:10.3390/pharmaceutics14071416)
Supplement: Supplementary file 1 [file pharmaceutics-14-01416-s001.zip › pharmaceutics-1783041-supplementary.pdf]

Supplementary Table S1. Quantitative PCR Primer Sequences

Gene	Orientation	Primer Sequence
SmSULT	Forward	5’-ATT GGA TGG TTA CAT AGC AAC TAC -3’
	Reverse	5’-CCA TGG ATC ATT TGA TTT GGG T -3’
ShSULT	Forward	5’- ATA GCT ACA ACA GAT CTA CCA TCA -3’
	Reverse	5’- TAA GTT TCC ATG GAT CCG TAG AT-3’
SjSULT	Forward	5’- TGATTGGTTGACAAGTTTTCG-3’
	Reverse	5’- CACTAAAAGACGTTCGGATGG-3’
SmGAPDH	Forward	5’- GTG AAA GAG ATC CAG CAA ACA T -3’
	Reverse	5’- ATA TGA GCC TGA GCT TTA TCA ATG-3’
ShGAPDH	Forward	5’- GAT CAA ATT AAG GCT GTG GTC A -3’
	Reverse	5’- CCA AAC TCA TTA TCG TAC CAT GAA -3’
SjGAPDH	Forward	5’- TCA GCT CAG ACT TTA TTG GAT GTA -3’
	Reverse	5’- TTG TCG TAC CAT GAA ACC AGT –3’
SmACTIN	Forward	5’- TGTTGTTGATAATGGATCAGGGA-3’
	Reverse	5’- CAGTTCGTCACAATACCGTG-3’
ShACTIN	Forward	5’- GACGAAGAAGTTCAAGCCCT-3’
	Reverse	5’- CGTGTTCGATTGGGTATTTCAG-3’
SjACTIN	Forward	5’- AGAGCTGTATTCCCTTCCATC-3’

	Reverse	5’- TCTTCTCCATATCATCCCAGTTTG-3’
SmTUBULIN	Forward	5’- CGGAATGGGAACACTACTCA-3’
	Reverse	5’- GACGACTGTATCAGAGACCTTAG-3’
ShTUBULIN	Forward	5’- TTGGTTGATTCCGTCTTAGATGT-3’
	Reverse	5’- TGTCAGATACTTTAGGCGATGG-3’
SjTUBULIN	Forward	5’- AATCAAATTGGTGCTAAGTTCTGG-3’
	Reverse	5’- CGTACACTGTCCATAGTTCCC-3’

Supplementary Table S2. Digital PCR Primer Sets

Gene	Orientation	Primer Sequence
SmSULT	Forward	5’-ATT GGA TGG TTA CAT AGC AAC TAC -3’
	Reverse	5’-CCA TGG ATC ATT TGA TTT GGG T -3’
ShSULT	Forward	5’- ATA GCT ACA ACA GAT CTA CCA TCA -3’
	Reverse	5’- TAA GTT TCC ATG GAT CCG TAG AT-3’
SjSULT	Forward	5’- TGATTGGTTGACAAGTTTTCG-3’
	Reverse	5’- CACTAAAAGACGTTCGGATGG-3’

Supplementary Table S3: SULT T7 Primer Sequences

Gene	Orientation	Primer Sequence	Size
SmSULT	Forward	5'- (T7) TCT CAG CTG GTC TAC CGA GAA-3'	591 bp sequence nt 41-606
	Reverse	5'- (T7)TCCCAACCATCACCAAGACG-3'	
ShSULT	Forward	5'- (T7)GGCCTACCAAGAACAGGTACAA-3'	569 bp sequence nt 85-654
	Reverse	5'- (T7)TGATTCCCAACCATCACCAAG-3'	
SjSULT	Forward	5'- (T7)GATACCTATAAAGAAGAAGTAGATAAAGTC-3'	197 bp sequence nt 436-633
	Reverse	5'- (T7)AGA CGT TCG GAT GT ACA -3'	

Example:
Fold change of *S. mansoni* SULT expression relative to *S. haematobium or S.japonicum* SULT expression

$$= \frac{E_{SmGAPDH}^{(Ct_{SmGAPDH})} \div E_{SmSULT}^{(Ct_{SmSULT})}}{E_{ShGAPDH}^{(Ct_{ShGAPDH})} \div E_{ShSULT}^{(Ct_{ShSULT})}}$$

Supplementary Figure S1. Formula for Cross Species *SULT* Transcript Comparison. The method above is algebraically equivalent to the ΔΔCt method for determining relative transcript quantities. In the example given, the fold change of *S. mansoni SULT* expression is determined relative to *S. haematobium or S. japonicum SULT*. $E_{SxGAPDH}$, $E_{SxActin}$ & E_{SxTub}: Efficiency of Internal Reference. E_{SxSULT} : Efficiency of SULT Primers. $Ct_{SxGAPDH}$, $Ct_{SxActin}$ & Ct_{SxTub}: Critical threshold of Internal Reference Primers. Ct_{SxSULT}: Critical threshold of SULT Primers. x = m,h, or j (*S. mansoni, S. haematobium, or S. japonicum*).

Video S1. Fluorescence *in situ* hybridization of *SmSULT*.

Head of *S. mansoni* male. Please see attached video.

Cells positive for *SmSULT* stained positive for TAMRA-Tyramide, magenta. DAPI (blue) was used at a final concentration of 1 ug/mL to stain cell nuclei. FITC-positive cells are *Schistosoma* stem cells, positive for *Schistosoma histone H2B* [30, 31]. *SmSULT* and *H2B* probes were used at a final concentration of 150 ng/mL. FISH was performed on whole worms. This picture is represented of 4 male worms.

h2b_sult_MG copy.mov
